# Bilateral septic acromioclavicular arthritis in a farm worker with high suspicion of zoonotic disease: a case report

**DOI:** 10.1007/s00132-024-04593-5

**Published:** 2024-12-23

**Authors:** K. A. Barlow, J. Gütler

**Affiliations:** 1 Orthopaedics Department, University Childrens Hospital Basel, Basel, Switzerland; 2 Orthopaedics and Traumatology Department, Kantonsspital Zug, Baar, Switzerland

**Keywords:** Septic arthritis, Acromioclavicular joint, Methicillin-susceptible *Staphylococcus aureus* (MSSA), Shoulder infection, Septische Arthritis, Schultereckgelenk, Methicillinsensibler Staphylococcus aureus (MSSA), Infektion der Schulter

## Abstract

**Introduction:**

We report the case of a 54-year-old male with the rare entity of bilateral septic acromioclavicular (AC) arthritis with osteomyelitis of the lateral clavicle with methicillin-susceptible *Staphylococcus aureus* (MSSA). The glenohumeral joint was affected as well. The patient was immunocompetent with no history of diabetes or intravenous drug abuse. There is a high possibility of a transmission from farm animals.

**Material and methods:**

The case files including laboratory measurements, imaging and documentation were used to reassess the case and complete the case report. Regular clinical follow-up was done in our outpatient clinic. We compared our case with the literature and existing guidelines.

**Results:**

We report this rare entity of bilateral septic AC arthritis to ensure early diagnosis and proper treatment in the future. In comparison to the existing guidelines, we followed similar treatment concepts. The outcome of our patient was satisfactory despite a pre-existing rotary cuff tear, which might need further treatment in the future.

**Conclusion:**

This report of a rare case can help to understand this infectious disease in the future and help establish treatment guidelines. In farm workers, one should be highly suspicious of transmission through animals and establish a quick and effective treatment as we did in this case.

## Abstract (de)

**Einleitung:**

Berichtet wird über den Fall eines 54-jährigen Manns mit dem seltenen Krankheitsbild einer bilateralen septischen Arthritis des Akromioklavikulargelenks (AK) und Osteomyelitis der lateralen Klavikula mit methicillinsensiblem Staphylococcus aureus (MSSA). Auch das Glenohumeralgelenk war betroffen. Der Patient war immunkompetent und anamnestisch ohne Diabetes oder intravenösen Drogenabusus. Es bestand eine hohe Wahrscheinlichkeit für eine Übertragung ausgehend von Vieh.

**Material und Methoden:**

Die Unterlagen des Patienten einschließlich Laboruntersuchungen, Bildgebung und Dokumentation wurden neu beurteilt, um den Fallbericht zu vervollständigen. Regelmäßige klinische Verlaufskontrollen erfolgten in unserer Ambulanz. Wir verglichen unseren Fall mit der Literatur und vorliegenden Leitlinien.

**Ergebnisse:**

Wir berichten über diesen seltenen Fall einer bilateralen septischen AC-Arthritis, um zukünftig eine frühe Diagnose und geeignete Therapie sicherzustellen. Wir wendeten ähnliche Therapiekonzepte an wie in den bestehenden Leitlinien. Die Behandlungsergebnisse unseres Patienten waren zufriedenstellend trotz einer bereits zuvor bestehenden Rotatorenmanschettenruptur, die eine weitere Therapie erfordern könnte.

**Schlussfolgerung:**

Der Bericht über unseren seltenen Fall kann das Verständnis dieser Infektionskrankheit fördern und bei der Erstellung von Therapieleitlinien hilfreich sein. Bei MitarbeiterInnen in der Landwirtschaft sollte stets die Möglichkeit einer Übertragung durch Tiere bedacht werden und eine schnelle und wirksame Therapie eingeleitet werden, wie im hier beschriebenen Fall geschehen.

## Introduction

Septic arthritis in the shoulder joint is a medical emergency with a high morbidity and can have a severe impact on patientsʼ quality of life if not treated immediately and in a correct way. Septic arthritis of the acromioclavicular joint (AC joint) is a rare entity with under 40 cases reported in literature [1, 2]. It can be easily missed or misdiagnosed. Differential diagnoses are activated degenerative arthritis, subacromial impingement, frozen shoulder or other glenohumeral pathologies such as omarthritis. Septic arthritis of the AC joint can occur in patients with comorbidities such as diabetes, drug abuse or immunosuppression. We present a case with bilateral involvement in the following case report.

## Case report

A 54-year-old male presented to the emergency room at night with atraumatic right shoulder pain since the day before. The patient was initially seen by the medical resident on call. The patient reported heavy farm labor for the past three days. He saw his general practitioner (GP) who started treatment with prednisolone for an adhesive capsulitis and analgetics (metamizole) with no improvement. The patient also reported a history of shoulder pain and stiffness in the past that resolved spontaneously. He has no history of fever, immunosuppression, nicotine abuse or known diabetes.

**Fig. 1:**
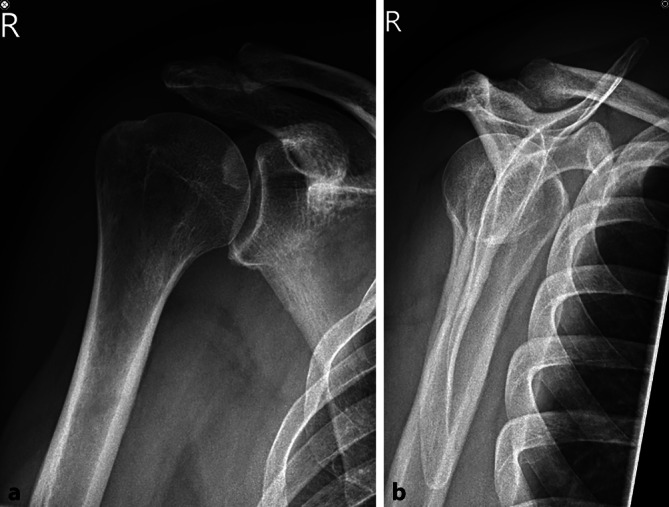
Initial X‑rays (right shoulder ap/Neer) with narrowing of AC joint space

**Fig. 2:**
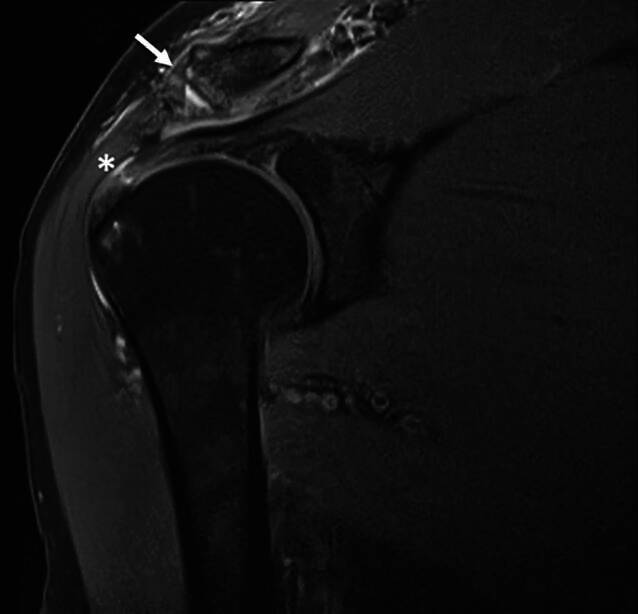
DW-MRI of the right shoulder showing effusion around the degenerative changed AC joint (arrow), asterisk: transmural lesion of supraspinatus tendon

**Fig. 3:**
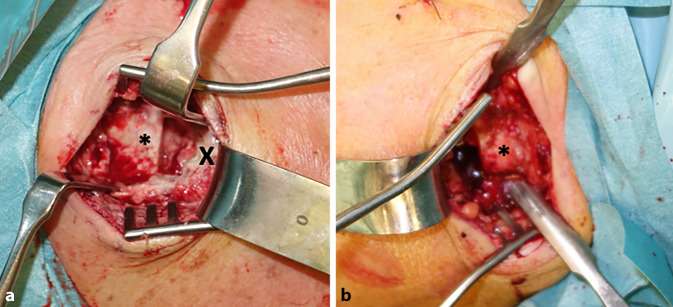
Fotograph from above of the right (**a**) and left (**b**) AC joint after lateral clavicle resection. Asterisk: lateral clavicle, X: pus

**Fig. 4:**
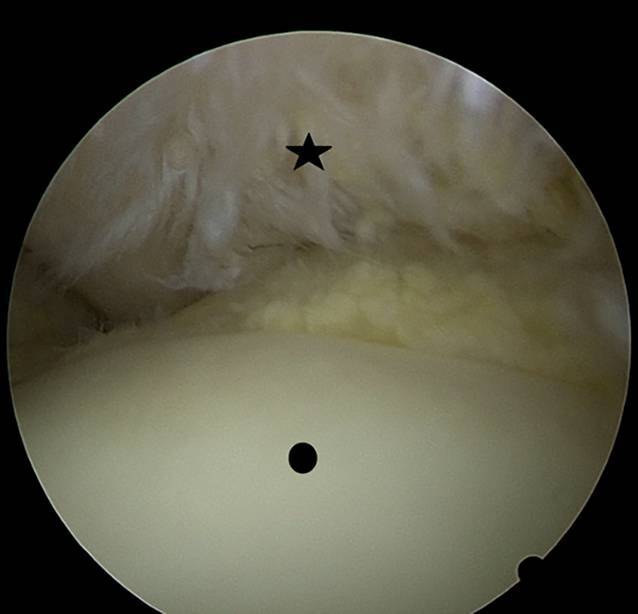
Degenerative rotator cuff tear (star) and humeral head (circle) intraoperative

**Fig. 5:**
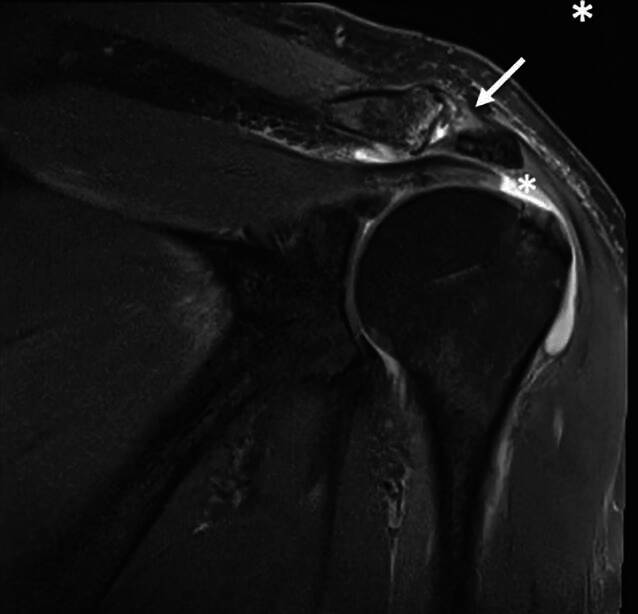
MRI of the left shoulder showing glenohumeral joint effusion (asterisk) and degenerative changes in the AC joint (arrow)

Clinically, the patient showed redness and swelling of the AC joint with tenderness to palpation. The active movement of his right shoulder was limited due to pain with anteversion of 30°, retroversion of 15°, abduction of 20° and external rotation of 15°. Strength testing was also limited due to pain but without lag signs. The X‑rays obtained showed degenerative changes in the AC joint with narrowing of the joint space Schweitzer grade three (Table 1 and Fig. 1). The patient was admitted to the orthopedic department for a consultation and possible joint infiltration with continuation of oral steroids. On the following morning, laboratory tests showed a C-reactive protein (CRP) of 71.4 mg/l (normal range < 5.0 mg/l) and leucocytes of 10.1 giga/l (normal range 4.0–10.0 giga/l). An ultrasound showed no glenohumeral effusion but fluid in the subacromial bursa. A diffusion-weighted magnetic resonance imaging (DW-MRI) was obtained that showed an arthritis of the AC joint with edema of the lateral clavicle as well as a fluid collection between the coracoid process and the anterior border of the subscapularis muscle (SSC, Fig. 2). Additionally, a degenerative transmural rupture of the supraspinatus muscle (SSP) was diagnosed. Due to exacerbating pain, the patient received a patient-controlled anesthesia pump with morphine. Follow-up laboratory tests on the next day showed an increasing CRP of 268.9 mg/l and leucocytes of 8.4 giga/l. The oral corticosteroids were stopped and the decision was made to take the patient to the operating room (OR) for resection of the lateral clavicle, debridement and irrigation of the AC joint and shoulder joint (glenohumeral and subacromial space) and to take multiple biopsies. The operation was started with open debridement of the AC joint and resection of the lateral clavicle which showed intra-articular pus with osteomyelitic changes of the lateral clavicle (Fig. 3). The open surgery was followed by arthroscopic debridement and synovectomy of the subacromial and glenohumeral spaces. The glenohumeral joint and subacromial space showed a synovialitis Gächter stage I [6] and a degenerative rotator cuff tear (Fig. 4). After taking samples for microbiology and histology, empirical i.v. antibiotic treatment was started with amoxicillin/clavulanic acid. In total, eight samples were taken from all three shoulder compartments. At the AC joint, samples were taken from bone as well as from soft tissue. Blood cultures were also obtained. They showed no bacterial growth after 15 days of incubation. The intraoperative samples showed penicillin and ampicillin resistant but methicillin-susceptible *Staphylococcus aureus* (Staph. aureus/MSSA). The antibiotic treatment was changed to oxacillin IV 2 g four times per day after receiving the results. The patient was planned for a second look surgery at two days postoperatively. He started complaining about left shoulder pain the day before, so that a punction of the shoulder compartments (glenohumeral, subacromial and AC joint) on the left was done after the second look surgery on the right side. After another two days, the glenohumeral fluid and the AC joint fluid of the left shoulder were positive for the same bacteria as on the right side, *S. aureus*. We decided to perform an open debridement and resection of the AC joint as well as an arthroscopic irrigation of the glenohumeral and subacromial spaces on the left side (Fig. 5). A preoperative MRI scan of the left shoulder showed less AC joint arthritis but a glenohumeral joint effusion with fluid in the subacromial space and a degenerative supraspinatus tear (Fig. 5). A third look with irrigation, debridement and biopsy sampling of all three compartments was done for the right shoulder and a second look subsequently for the left shoulder after another three days. There was no more growth of any bacteria in the cultures obtained.

**Table 1:** Grades of AC joint osteoarthritis, modified after Schweitzer et al. [4]

Grade	Radiologic features
0	No changes
1	Narrowing of joint space
2	Subchondral sclerosis, cartilage erosion
3	Cysts, osteophytes, heavy wear of cartilage

We continued IV antibiotics for a total of 2 weeks and oral antibiotics for a total of 8 weeks. The case was frequently discussed with colleagues from the infectious diseases department. The patient was seen in our outpatient clinic 8 weeks after discharge and 2 weeks after finishing the oral antibiotics. There were no clinical signs of persistent infection and the blood work showed normal infectious parameters (CRP 1.9 mg/l and leucocyte count 6.0 giga/l). The patient is still symptomatic for the pre-existing rotator cuff tear, which might need further treatment in the future.

Additionally, the blood sugar profile was measured and showed lightly elevated fasting blood sugar and HbA1C (fasting blood sugar: 6.9 mmol/l; HbA1c (Diabetes Control and Complications Trial (DCCT)/National Glycohemoglobin Standardization Program (NGSP) and HbA1c 6% (reference level: 4.8–5.9%)) which was interpreted as a side effect of the infection by our endocrinologists. A transthoracic echocardiogram showed no signs of endocarditis.

Interestingly, the patient reported, that two of the cows on his farm were diagnosed with a dug infection of Staph. aureus two months ago. The patient was milking the cows by hand at the time without protective gloves. He presented with multiple rhagades and calluses all over his hands as a manual worker. An antimicrobial suspectibility test (AST) was not obtained by the veterinarian at the time. The cows were treated with a combination of gentamicin/penicillin. We were able to acquire another sample of milk of one of the cows, that had previously had the infection to compare the bacteria. Unfortunately, it showed no bacterial growth; however, the cow was also not symptomatic anymore at the time the sample was taken.

## Discussion

We report this case of a rare condition of bilateral acromioclavicular joint arthritis. We highly suspect a transmission from farm animals which has not been reported in the literature before. It is important to get a full history and ask about the background of the patients to ensure a correct diagnosis and provide the best possible treatment. We wanted to draw attention to this rare entity again to ensure early recognition and proper treatment of similar patients in the future. Steinmetz et al. published a case series of three cases and developed a treatment algorithm [2]. They recommend an X‑ray and laboratory work-up as the first line diagnostics and an ultrasound or MRI if a septic arthritis is suspected. They also recommend surgical irrigation followed by selective antibiotic treatment for 4–6 weeks with consultation of the infectious diseases department. We followed a similar treatment regimen and saw a satisfactory result in our patient and therefore support their algorithm. The case reported showed a bilateral involvement of the AC joints. We found one other case [3] in the literature with a bilateral infection. The patient reported there was also diagnosed with a *Streptococcus pneumoniae* endocarditis. We therefore strongly recommend to rule out sepsis and involvement of other organs while treating patients with AC joint infection.

In the presented case, the initial administration of oral corticosteroids might have led to a more severe progression of the infectious disease; however, there are newer studies suggesting that corticosteroids have a protective effect on cartilage in septic arthritis in children as well as in animal models [5]. Farrow describes a positive effect of adding corticosteroids to antibiotic treatment in various studies in his review from 2015. He also stated that further studies are needed to better investigate the use of corticosteroids in the adult human population [5].

The patient presented had a pre-existing AC joint osteoarthritis grade III after Schweitzer et al. (Table 1; [4]). It is known that the pre-existing inflammatory process and chronic joint conditions increase the risk of septic arthritis, so this might have led to the manifestation of the infection in the AC joints in this patient.

## Conclusion

This report of a rare case can help to understand this infectious disease in the future and help establish treatment guidelines. In farm workers, one should be highly suspicious of transmission through animals and establish a quick and effective treatment as we did in this case.

## Abkürzungen

AC Gelenk: Akromioklaivulargelenk/​Schultereckgelenk
AC joint: Acromio-clavicular joint
AST: Antimicrobial suspectibility test
CRP: C-reaktive protein
DCCT: Diabetes Control and Complications Trial
DW-MRI: Diffusion weighted magnet resonance imaging
GP: General practitioner
MSSA: Methcillin-suspectible *Staphylococcus aureus*
NGSP: National Glycohemoglobin Standardization Program
SSC: subscapularis muscle
SSP: supraspinatus muscle

## Declarations

For this article no studies with human participants or animals were performed by any of the authors. All studies mentioned were in accordance with the ethical standards indicated in each case. For images or other information within the manuscript which identify the patient consent was obtained.
